# Perceptions of environmental changes and Lethargic crab disease among crab harvesters in a Brazilian coastal community

**DOI:** 10.1186/1746-4269-7-34

**Published:** 2011-11-16

**Authors:** Angélica MS Firmo, Mônica MP Tognella, Walter LO Có, Raynner RD Barboza, Rômulo RN Alves

**Affiliations:** 1 Departamento de Ciências Agrárias e Biológicas, Programa de Pós Graduação em Biodiversidade Tropical (Ecologia), Universidade Federal do Espírito Santo - UFES, São Mateus, Brazil; 2 Departamento de Biologia, Associação Educacional de Vitória - AEV, Vitória, Brazil; 3 Departamento de Biologia, Programa de Pós-Graduação em Ciências Biológicas (Zoologia) Universidade Estadual da Paraíba - UEPB, Paraíba, Brazil

**Keywords:** Crab harvesters, mangrove, ecological knowledge, Lethargic Crab Disease

## Abstract

**Background:**

Lethargic Crab Disease (LCD) has caused significant mortalities in the population of *Ucides cordatus *crabs in the Mucuri estuary in Bahia State, Brazil, and has brought social and economic problems to many crab-harvesting communities that depend on this natural resource. The present work examined the perceptions of members of a Brazilian crab harvesting community concerning environmental changes and the Lethargic Crab Disease.

**Methods:**

Field work was undertaken during the period between January and April/2009, with weekly or biweekly field excursions during which open and semi-structured interviews were held with local residents in the municipality of Mucuri, Bahia State, Brazil. A total of 23 individuals were interviewed, all of whom had at least 20 years of crab-collecting experience in the study region. Key-informants (more experienced crab harvesters) were selected among the interviewees using the "native specialist" criterion.

**Results:**

According to the collectors, LCD reached the Mucuri mangroves between 2004 and 2005, decimating almost all crab population in the area, and in 2007, 2008 and 2009 high mortalities of *U. cordatus *were again observed as a result of recurrences of this disease in the region. In addition to LCD, crabs were also suffering great stock reductions due to habitat degradation caused by deforestation, landfills, sewage effluents, domestic and industrial wastes and the introduction of exotic fish in the Mucuri River estuary. The harvesting community was found to have significant ecological knowledge about the functioning of mangrove swamp ecology, the biology of crabs, and the mass mortality that directly affected the economy of this community, and this information was largely in accordance with scientific knowledge.

**Conclusions:**

The study of traditional knowledge makes it possible to better understand human interactions with the environment and aids in the elaboration of appropriate strategies for natural resource conservation.

## Background

Mangrove forests are highly productive ecosystems found along the coast of Brazil that provide valuable resources such as timber, medicinal products, natural dyes, fish, crustaceans, and mollusks, as well as environmental services [1-6]. Brachyura crabs are a major economic resource for the coastal dwellers of northeastern Brazil, as either subsistence economic items or for direct consumption. The main species commercialized are blue land crabs (*Cardisoma guanhumi *(Latreille, 1825), callinectes crabs (*Callinectes *spp.), and mangrove land crabs (*Ucides cordatus *Linnaeus, 1763). The mangrove land crab is the most heavily harvested species, and therefore of particular relevance to people living in the surrounding mangrove areas [7].

*Ucides cordatus *inhabits in individual burrows about 120 cm deep that are dug under mangrove trees [8]. Studies undertaken in northern Brazil have determined that adult mangrove crabs have few natural predators, but these included the crab-eating raccoon (*Procyon cancrivorous *(Cuvier, 1798), monkeys, and hawks [9]. High predation pressure is exerted on *U. cordatus *by humans, however, who harvest this species for direct consumption [10] and principally for commercialization. The harvesting of *U. cordatus *has significant socioeconomic importance in northeastern Brazil and involves many local residents who benefit both directly and indirectly from commerce involving this species [7,11].

Massive mortalities of *U. cordatus *have been reported by crab harvesters and biologists since 1997 throughout northeastern Brazil, from Ceará to Espírito Santo State [12,13] that have decimated local stocks (reaching 84% reductions, according to interviews with crab-harvesters) [7]. This mortality has generated considerable concern among specialists in regards to resulting environmental and socioeconomic impacts [7].

Many investigations were undertaken to determine the causative agent of this mass mortality, until Boeger et al [12] conclude that the causative agent as an *Exophiala *species of fungus. This disease, which is specific to the crab species *Ucides cordatus*, is the first known disease caused by a fungus in crustaceans [14]. Due to the fact that numerous symptoms were shared by many crabs in areas with high mortality rates, such as lethargy, deficient motor control, and an incapacity to return to an upright position, this disease was given the name Lethargic Crab Disease (LCD) [15].

Decreases in crustacean populations have created social problems in the communities surroundings mangrove areas and seriously affected the economic welfare of the poor people who depended upon crab harvesting for their livelihood. The life of crab harvesters is intimately linked to ecological processes and cycles, and their daily work with other natural resources has helped them develop harvesting strategies that maximize their crab harvesting efficiency. The understanding these local residents have of the ecology of *U. cordatus *is an important factor in the use of this natural resource [11,16], and the present research sought to characterize the traditional knowledge of these crab harvesters regarding environmental changes in the mangrove forests of the Mucuri River estuary, Bahia State, in northeastern Brazil and regarding Lethargic Crab Disease in the same region.

## Methods

### Study area

**Figure 1:**
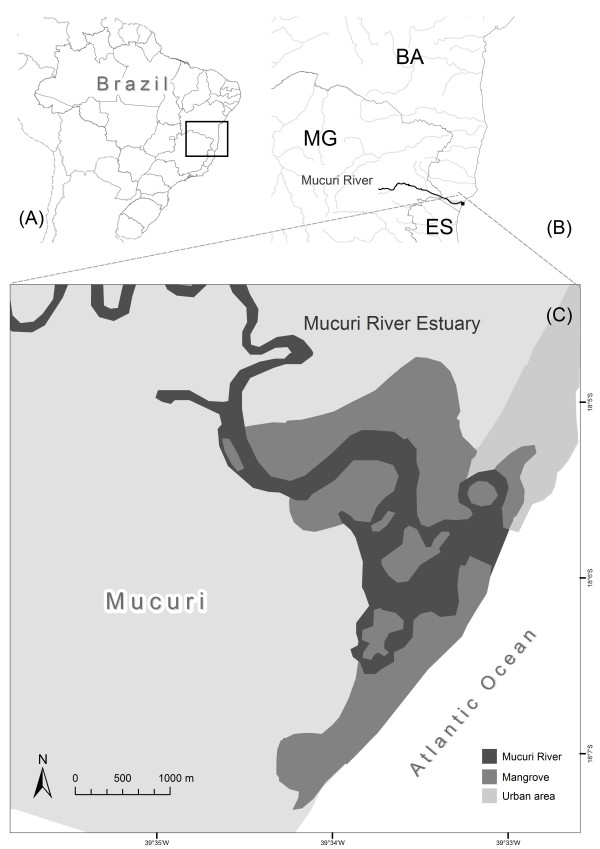
**Map of the Mucuri River estuary, Bahia state, Brazil**.

**Figure 2:**
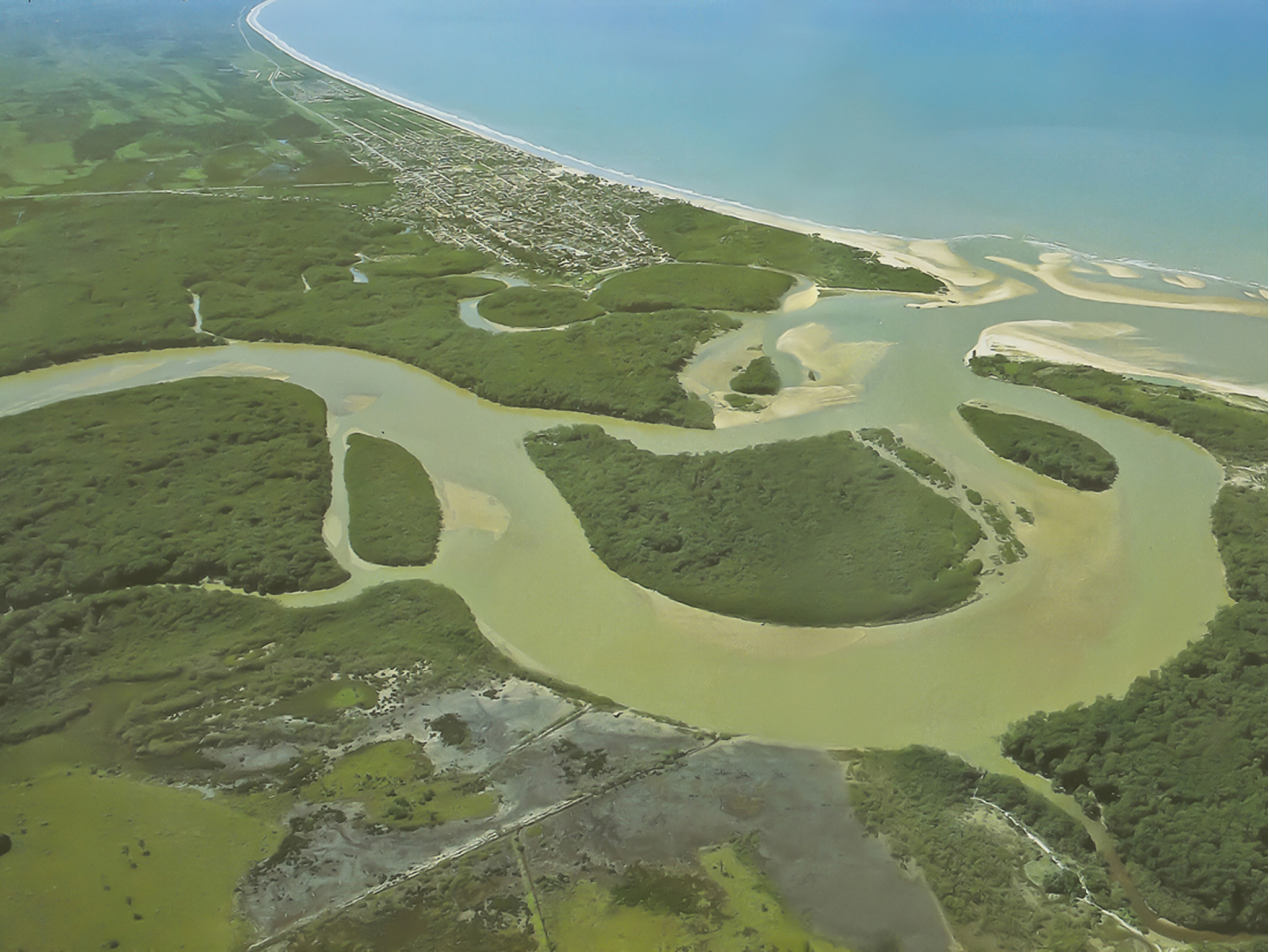
**Aerial view of Mucuri River estuary and the municipality of Mucuri**.

The present research was carried out in the mangrove estuary of Mucuri River located in the municipality of Mucuri, Bahia State, in northeastern Brazil (17°51'00" - 18°05'17" S and 39°33'14"- 40°00" W) (Figure 1). The entire area of influence of the Mucuri River is contained within the Costa Dourada Permanent Protection Area, under control of the Brazilian Institute for the Environment and Natural Resources - IBAMA (Figure 2). The estuary contains large extensions of mangrove forests, islands, and islets (these latter formed by sandy-clay-loam banks). The most common trees found in the mangrove forests are *Rhizophora mangle, Avicennia germinans, Avicennia schaueriana*, and *Laguncularia racemosa *[17].

### Data collection and analyses

Our research was undertaken between January and April/2009, with weekly or biweekly field excursions. A community meeting was held with crab harvesters before initiating the actual fieldwork to inform them about the aims of our study, our research methods, and to solicit their participation in the investigations. Qualitative methods were used to obtain information about environmental changes that have occurred in the mangrove forest, crab harvesting, and the local perception of Lethargic Crab Disease. We did not adopt a formal approach using interview consent forms, due to the poor level of organization within the fishing community and the high illiteracy rate.

Open and semi-structured interviews were conducted with 23 people, and additional interviewees were chosen by using the "snowball technique" [18,19], based on information provided by local specialists. Interviews were conducted on a one-on-one basis. Key-informants (more experienced crab harvesters) were selected from among the interviewees using the criterion of "native specialists" - people who consider themselves, and are considered by the community, as culturally competent in this area [20,21].

All of the interviewees harvested crabs as their main economic activity (active or retired) and had at least 20 years of experience in the study region. Each collector was interviewed individually, time limit for the interviews (which generally lasted between 1 and 3 hours each). Interviews consisted of asking crab harvesters about two subjects: A) to describe the community's perception of its relationship to the mangrove forests (to determine their views of the degradation and conservation of the mangrove forests and the management of natural resources); B) to describe the community's perception of the Lethargic Crab Disease and the socioeconomic problems resulting from it. These questions were presented to the crab harvesters using a standardized questionnaire with 16 questions; the details of the interviews were recorded manually and/or by using a voice recorder. The transcriptions were made with a full awareness of the need to be faithful to the interviewees manner of expression.

The interviews were always preceded by the interviewer's identification with a brief explanation about the purpose of work and an informed consent and permission for publication of the images were given by those interviewed. Other techniques used included direct observations (accompanying the crab harvesters individually during their daily activities) and "guided tours" - two integrate the researchers and interviewees and to experience their routine activities in their natural environment.

In addition to the interviews with the crab harvesters, an ethno-mapping of the Mucuri River estuary mangrove forests was undertaken using aerial photographs and maps instead of illustrations and drawings made by the crab harvesters. This mapping examined the perceptions of local informants concerning the locations of local natural resources [22], as we asked them to identify the different areas of the mangrove forests (and to correctly name them) and to indicate which areas were most affected by the crab disease and the most vulnerable environments.

## Results

### Perceptions of environmental changes

When asked about their relationships with the mangrove forests, the interviewees reported being directly linked with those environments and they considered the estuary to be one of the most important features in their lives - especially since their economic survival was almost completely dependent on crab harvesting. Nineteen of the interviewees reported that they appreciated (were proud of) their profession as crab harvesters and that they intended to continue in that occupation. All of them, however, reported that the mass mortality that had decimated the crab stocks in the Mucuri River estuary was making it almost impossible to continue in their profession. Many crab harvesters were very unhappy with the situation and doubted their ability to continue working there if the land crab stocks were not restored.

When asked about how to best protect the mangrove forests and the mangrove land crabs, the interviewees gave different responses. Some replied that they would like to act as monitors to prohibit capture during the reproductive period, while others stated that people should stop destroying the mangrove forests and restore the environment, and avoid polluting the river estuary. All of the crab harvesters appeal for financial support from the government so that they could afford to stop gathering crabs during their reproductive and ecdysis periods. Financial support is in fact provided to fishermen and crab harvesters in Brazil during the animals' reproductive periods; however, the crab harvesters in this particular municipality do not have any registered association so they cannot receive any financial support.

All of the interviewees affirmed that before the appearance of the disease they were only authorized to capture *Ucides cordatus *more than 6.0 cm. In Brazil, harvesting mangrove land crab is regulated by Normative Instruction n° 34 of March 24, 2003 issued by the Instituto Brasileiro do Meio Ambiente e dos Recursos Naturais Renováveis (IBAMA), which prohibits taking female crabs during the period between December 1 and May 31, or capturing males or females less than 6.0 cm long.

**Figure 3:**
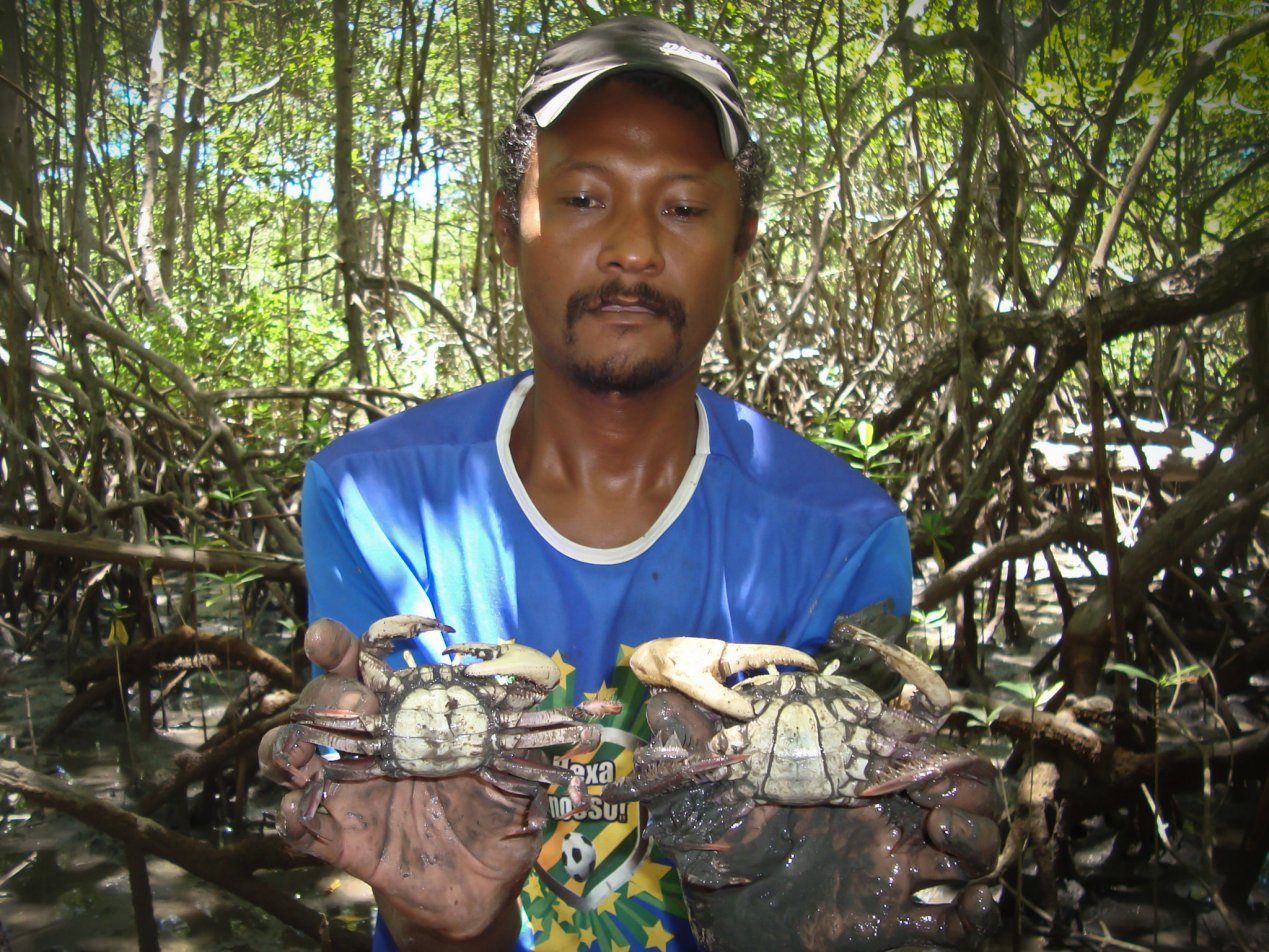
**Collector showing the size and sexual dimorphism of the land crab species (*Ucides cordatus*)**.

The interviewees reported that they were able to distinguish males from females by their tracks at the burrow entrances. The fishermen call the female crabs 'candurua' (a corruption of the word for 'female kangaroo' in informal Brazilian Portuguese) because they carry their egg masses on their abdomens. The females do not have any hair on their legs and leave deep thin tracks at the burrow entrances; the hairy legs of males leave relatively wide and shallow tracks (Figure 3).

All of the interviewees reported that harvesting mangrove land crabs was their only (or principal) economic activity. Only one interviewee reported harvesting blue land crabs (*Cardisoma guanhumi*) as well as mangrove land crab, and two crab harvesters were also fishermen.

**Figure 4:**
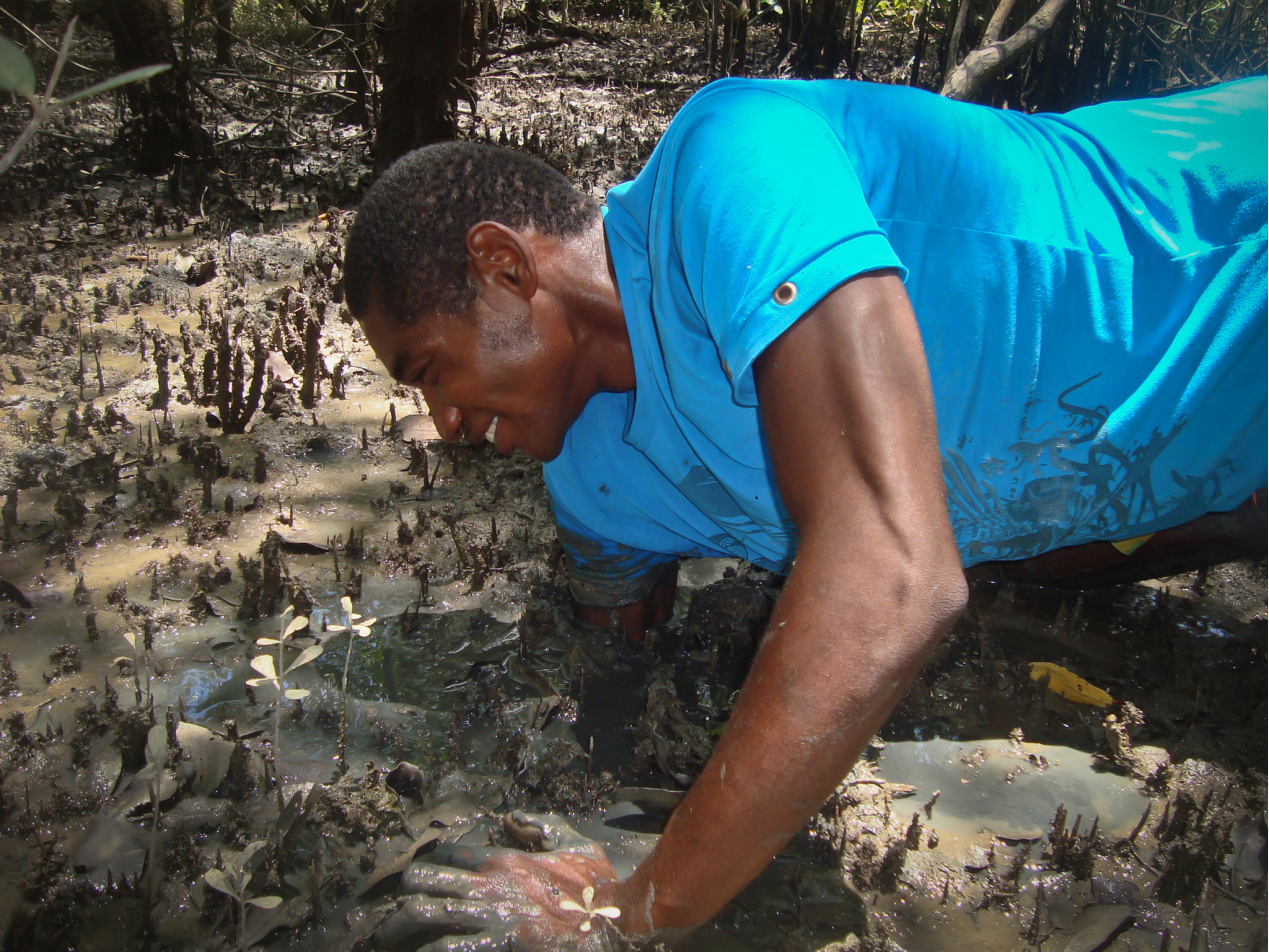
**Crab collector demonstrating an arm collection technique: the gatherers put their entire arms in the mud in order to catch the crab of their burrows**.

Among the interviewees, 21 said that they did not have any other economic activity besides crab gathering. When asked about the collection techniques they used for capturing mangrove land crabs, all of the interviewees claimed to use the manual collection technique. This technique requires putting one's entire arm into the burrow and grabbing the animal by the dorsal portion of its carapace (Figure 4) and then pulling it out in a lateral position. Only one interviewee reported using a curved iron tool to pull the crab from its burrow.

There is another technique called "redinha" used in crab harvesting that employs in amorphous mass of thin plastic mesh that is placed at the mouth of the burrow to entangle the crabs and facilitate their capture. This technique is considered predatory (and illegal) by the crab harvesters because it indiscriminately traps adults and juveniles, males and ovigerous females; these snares are often lost and can cause the death of other crabs as they pollute the mangrove area.

Consequently, crab harvesters are well aware of the use of "redinhas" in the region, although none of the interviewees claimed to use them. When asked about the best period for collecting crabs all of the interviewees indicated that the summer and reproductive season were best (between December and March). The crabs all leave their burrows during this period to copulate and are easily captured as they lose their defensive and directional instincts.

All of the interviewees also felt that the exotic fish being farmed in the Mucuri River estuary (e.g. Tucunaré peacock bass (*Cichla *sp.) and the African sharpthooth catfish (*Clarias gariepinus*) were related to the decreases in crab populations. The crab harvesters said that these fish were introduced into ponds along the Mucuri River and that they often escape to the estuary during floods, where they feed on crab larvae, thus diminishing crab populations.

### Perceptions of Lethargic Crab Disease

When asked about diseases that could be causing massive crap die offs, the interviewees all confirmed that they knew about the disease currently affecting the crustacean. They stated that there is currently a mortality rate of approximately 50% when compared to the period just prior to the arrival of the disease. Interestingly, none of the crab harvesters knew the official name of this disease.

According to most of the interviewees (about 70%), the disease first appeared in the Mucuri River estuary between September and October/2004 and resulted in very high mortality rates in 2005, and all of the crab harvesters said that the disease was still affecting the area. This was confirmed by the authors during the field work phase of this project, as many did mangrove land crabs were found, while others presented clinical signs of LCD. The crab harvesters reported that only small and young crabs could be found in the area. They also stated that there were large die offs of *U. cordatus *in 2007, 2008 and 2009 as a result of recurrences of this disease in the region.

According to the interviewees, there are periods with high mortality rates and others in which the disease relents a bit and crab populations increase.

**Figure 5:**
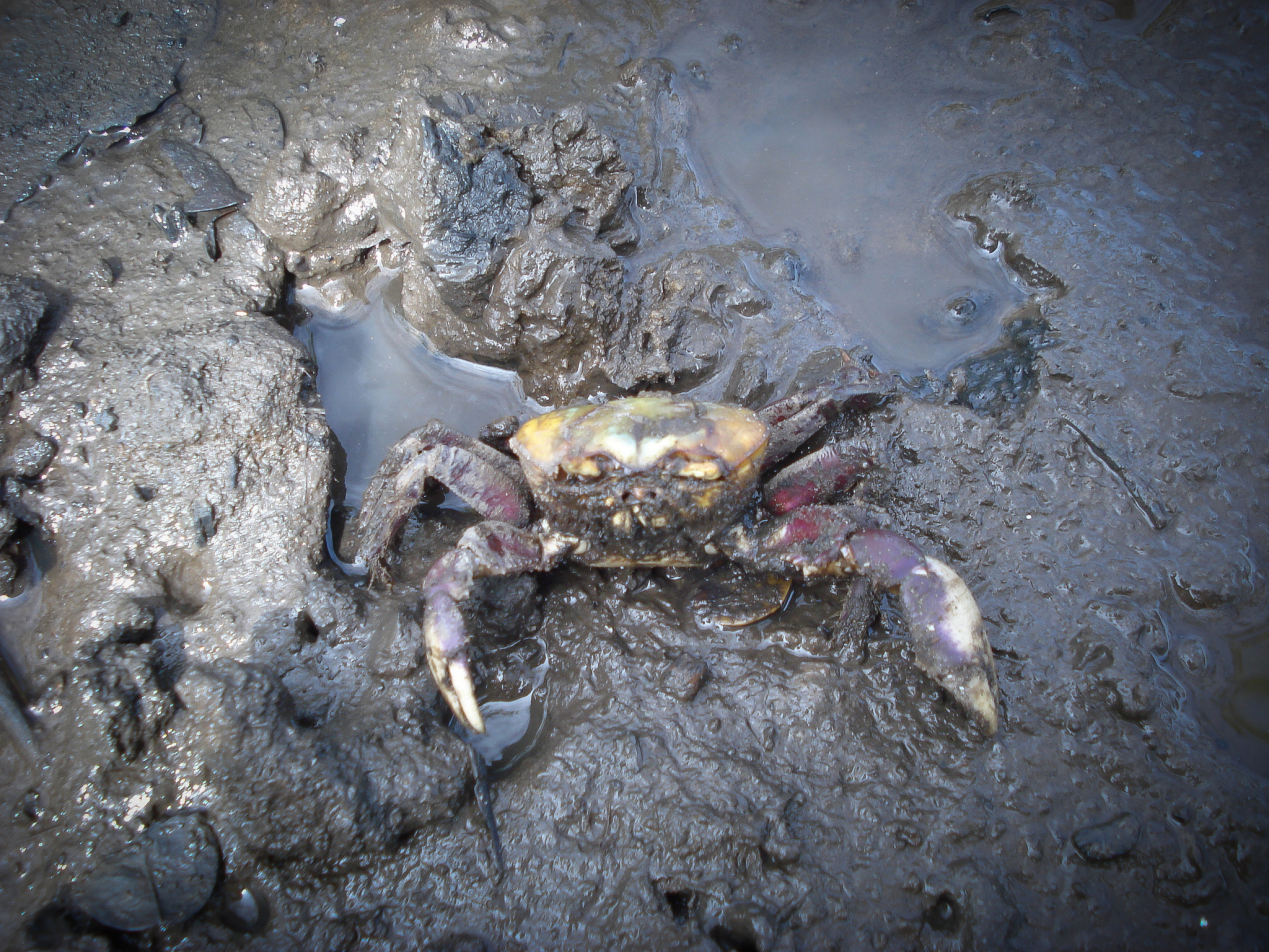
***Ucides cordatus *specimen captured probably presenting the signs of LCD disease**.

**Figure 6:**
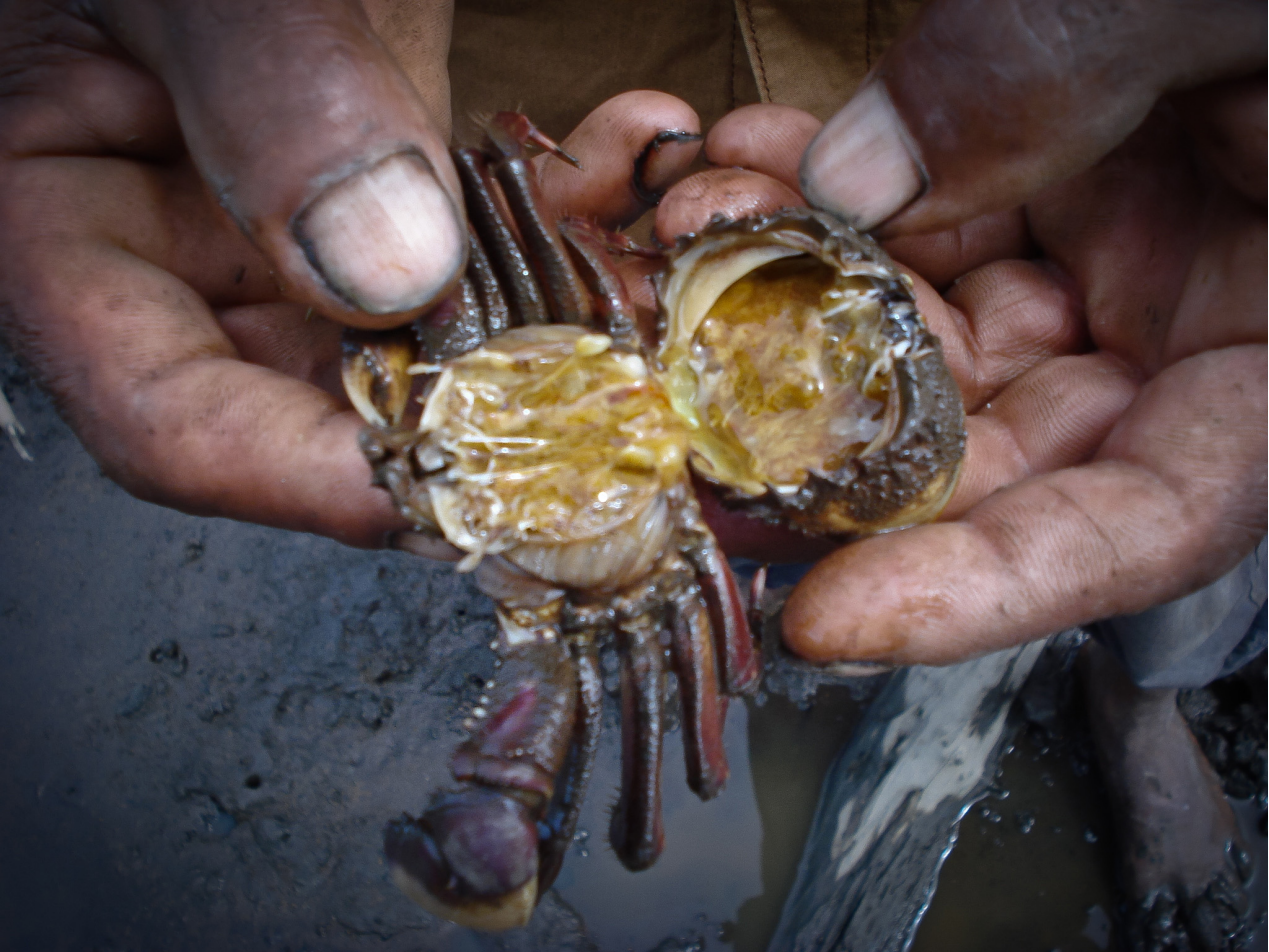
**Crab collector showing the internal organ of a dead crab presumably by lethargic crab disease**.

Regarding the clinical signs noted by the crab harvesters, all of them cited lethargy, differences in colors and textures of the internal organs in infected individuals, and the presence of foam coming from the crustacean's mouth region. According to the crab harvesters, animals contaminated by LCD appear "dirty", which is notable, for even though they live in the mud the crustaceans always appear groomed and clean; the crabs also lose their reflexes and will remain still with their eyes and claws drooping (Figure 5). An interesting phenomenon reported by all of the interviewees was that the disease leads to changes in the behavior of the mangrove land crabs, and they show exaggerated defensive behaviors, staying inside their burrows for long periods of time and covering their tunnels even though they were not undergoing ecdysis. When asked to identify the areas of the Mucuri River estuary that were most affected by the disease and that had the highest mortality rates, the interviewees indicated the areas closest to a paper factory that was discharging its wastes in the river (Figure 6).

The major (or only) economic resource of many local families is provided by crab harvesting, and since these people did not receive any assistance that could minimize the economic shock resulting from the scarcity of crabs, significant socioeconomic impacts were felt. Many crab harvesters were unhappy with their current situation and indicated that they would consider abandoning the profession if the stocks of mangrove land crabs did not rebound. Another problem that arose after the appearance of LCD was the noncompliance with environmental laws that regulate crab harvesting activities. The fishermen pointed out that it was no longer possible to find crabs larger than the legal limit of 6.0 cm; they also noted that it was impossible to respect the reproductive season of the crab (when the animals were more available).

Many crab harvesters reported that previously to the outbreak of the disease they could harvest between 75 and 100 crabs in a single day, but this number was drastically reduced to 25 crabs a day with the appearance of LCD. The crab harvesters are well aware that this situation may lead to even greater decreases in crab populations, but they cite their need to survive.

## Discussion

In terms of the relationships of crab harvesters with the mangrove swamp, we were able to diagnose a direct link between the estuary and the survival of the local human populations. This relationship was very evident in the present study, as it had been in other previous field investigations [7,23,24]. Most of the crab harvesters built their lives around coastal areas, estuaries and mangroves, and the knowledge passed to them by their ancestors, together with their own experiences, helped them to work and survive in mangrove swamps and to be recognized as members of a traditional population. Diegues [25] noted that knowledge about the cycles and dynamics of nature that have been passed down from generation to generation represent one of the principal components that characterize the traditional communities and allows these people to develop and use effective resource management strategies.

We noted in this study that crab harvesters differentiate between male and female mangrove land crabs. This finding was similar to that of other authors [26] who studied environmental perceptions among crab harvesters in Paraiba State in northeastern Brazil - which suggests that this information is endemic to traditional coastal communities throughout the country. This type of information is very important in terms of the conservation of mangrove land crabs, as this species is subjected to high predation pressure rates and the harvesting of females is pivotal to maintaining viable crab populations. If this behavior were recognized and encouraged, the fishermen would become partners to state and federal agencies in public conservation policies.

Regional studies should be undertaken to better orient governmental institutions in adopting conservation programs and regulations. This type of orientation could be very important as only mature males can currently be captured - and this will alter the proportions of males and females and leads to sexually unbalanced populations. Accordingly, studies focused on separate regions could examine male and female captures in different portions to determine optimal management and conservation techniques for this crustacean.

Regarding the local knowledge of the LCD disease affecting *U. cordatus*, most interviewees reported being aware of this disease, although none of them could name it. Interestingly, while the interviewees could not name the disease that was infecting the crabs in the region, all of them could identify the major clinical symptoms of LCD. This inability to correctly name the disease is almost certainly due to the fact that it was only recognized and named by Boeger et al [15] (in reference to the lethargy demonstrated by infected crustaceans). These authors provided robust evidence suggesting that LCD is caused by an anamorph fungus and phylogenetic analyses have confirmed the diagnosis of the LCD fungus as an ascomycete (fungus of the phylum Ascomycota), and they suggest a close relationship with members of the subphylum Pezizomycotina. Direct cultures of tissue from sick crabs, and subsequent isolation and purification, have identified the causative agent as an Exophiala species of fungus that affects the tissues and/or the hemal lacunae of the epidermis, connective tissue, hepatopancreas, heart, thoracic ganglion and associated nerve fibers, gills, and intestines [12]. Orélis-Ribeiro et al [27], fulfilling the rigorous Koch postulates, concluded that the *Exophiala cancerae *species is the causative agent of LCD. His results support that the fungal agent is pathogenic and upheld the hypothesis of Boeger et al [12,15] that associated this fungus with LCD and the mass die offs of crabs.

High rates of crab mortality were first noted in Brazil in 1997 and precipitated profound alterations in coastal communities that depended on this crustacean resource [7]. The mangrove swamps in the states of Bahia and Pernambuco suffered reductions of 84% and 97.6% in their *U. cordatus *stocks respectively. These reductions were directly associated with the massive die offs that seriously affected local fishing communities and led to social and economic problems in the region. In addition to LCD, crab stocks are also being impacted by habitat degradation as mangrove areas forests are subjected to deforestation and detrimental activities such as carciniculture and waste and garbage disposal [28]. This view is reinforced by other authors [7] who reported that *U. cordatus *harvesters in northeastern Brazil associated the occurrence of LCD with pesticide use in sugar cane plantations near mangrove forests. But there is still considerable uncertainty surrounding the origin and dissemination of this disease.

According to a report published by the Goiamun Institute [29,30], technical visits/field studies were undertaken in mangrove forests in the municipalities of Nova Viçosa and Mucuri (Bahia State, Brazil) to follow the evolution of this disease and the massive die offs of mangrove land crabs. This report indicated that no burrows of adult or sub-adult crabs could be found, leading to the conclusion that crabs in these age classes were heavily affected. These results are in agreement with the observations of the interviewees in the present study, as they only reported encountering small crabs, probably young ones. Schmidt [30] study the effects of one LCD event on *U. cordatus *in southern Bahia, and observed that most of the crabs left their burrows before dying and that individuals of all size classes were affected, although the larger animals, occupying zones that were more heavily flooded, appeared to be most affected by the mass die offs - and the greatest reductions in population density occurred in zones occupied by the mangrove tree *Rhizophora mangle*, indicating that the causative agent was in some way associated with water.

These observations lead us to presume a greater vulnerability of adult crabs to this disease, or that some aspect of the infectious chain is more relevant in adult crustaceans.

Mangrove land crab populations do not disappear completely, leading scientists to conclude that some individuals are naturally resistant to the disease. Researchers likewise observed that other crab species (such as *Goniopsis *sp., *Uca *spp., and the blue land crab *Cardisoma guanhumi*) were not affected by the disease. As most of the interviewees only harvested *U. cordatus*, we could not confirm this particular aspect of the disease.

The defensive behavior of crabs presenting clinical signs of LCD was highlighted by all of the interviewees. This behavioral change was characterized by the animals covering their burrows and staying in them for long periods of time. This behavior was also reported by Schmidt [30], who reported massive mortality rates in *U. cordatus *populations in the mangrove forests of Caravelas, Bahia State, Brazil. Based on these observations, it is possible to conclude that these behavioral changes are associated with avoidance of exposure to predators, as the crabs are much more vulnerable when manifesting clinical signs of LCD.

Many crab harvesters reported environmental changes associated with the introduction of exotic animal species into the estuary, and exotic fish species are known to affect the structure and functioning of aquatic environments [31]. The introduction of exotic fish species can cause many ecological changes affecting biodiversity through biological mechanisms such as hybridization and disease [32] and could, in this particular case, have contributed to crab mortality.

When asked which areas of the Mucuri River estuary mangrove forests were most affected by the disease and presented the highest crab mortality rates, the interviewees indicated sites close to the effluent pipes of a paper factory - leading the crab harvesters to associate the death of the crustaceans with the discharged waste. We must stress, however, that this is the point of view of the crab harvesters, and the direct cause of the disease affecting the Mucuri River estuary mangrove land crabs has yet to be fully determined.

Traditional knowledge can be critical to establishing sound policies regulating the sustainable exploitation of natural resources [7,11]. Brazilian governmental offices responsible for environmental monitoring and for controlling *U. cordatus *harvesting have not made a practice of considering local ecological knowledge - which could explain the generally low effectiveness of their policies [33]. The perceptions of local populations can and should be used in formulating the policies of regulatory agencies. The contributions of these communities are essential to the sustainable management of local resources, and need to be incorporated into decision making processes.

## Conclusions

Additional ethnoecology and ethnobiology studies of *Ucides cordatus *are needed, for the folk knowledge retained by traditional communities concerning the natural resources they come into contact with on a daily basis can benefit efforts to conserve and preserve regional biodiversity [7,11,21,24,25,33-42].

*U. cordatus *gatherers have developed an intimate knowledge of the natural history of this species. The unique nature of this local knowledge demonstrates the need for considering input from these populations in the implementation of management plans of coastal mangrove ecosystems, and for understanding local crab stocks and their population dynamics. This kind of information can be used for establishing extractive reserves, for delimiting the harvest season, and for establishing protected areas where animal species can safely reproduce and maintain their stocks.

It has become evident that efforts directed towards the preservation of native species should employ gradual and integrated approaches, coupled with monitoring of the communities involved, and these communities must be inserted into programs of environmental education and social assistance.

## Competing interests

The authors declare that they have no competing interests.

## Authors' contributions

AMSF - Writing of the manuscript, literature survey and interpretation; AMSF, MMPT, WLOC, RRDB and RRNA - Ethnozoological data, literature survey and interpretation; AMSF and MMPT - Analysis of taxonomic aspects. All authors read and approved the final manuscript.
